# Vomiting of Pregnancy

**Published:** 1867-09

**Authors:** 


The following excerpta we make from the Druggists' Circular:

*Vomiting of Pregnancy.*

In a recent discussion on the sickness of Pregnant Women, published in the *Union Médicale*, M. Gros reported a case in which this symptom had continued for a long time, reducing the patient to extreme emaciation. He administered pepsine in the dose of fifty centigrammes (about eight grains) before each meal, with complete relief. Many other remedies had been previously tried without the least benefit.

The efficacy of this remedy in these cases was substanciated by Drs. Duhomme, Piogey, and Labbé. It was remarked that the action of this remedy is somewhat uncertain, owing to its liability to change. It should not be administered in too hot a vehicle, as a high temperature destroys its efficacy. Another objection to its common use is its great price.

Dr. Dufour said that in many cases he had found chlorohydic acid an equally efficient remedy, and not liable to the objections which exist against pepsine; he had found it to be almost equally successful in these cases.—*Boston Med. and Surg. Journal.*

